# Supplementary material for: Analysing quantized resistance behaviour in graphene Corbino p-n junction devices
Source: J Phys D Appl Phys. Author manuscript; Available in PMC 2021 Jan 1. (PMC7431976; doi:10.1088/1361-6463/ab83bb)
Supplement: Rigosi_JPD_SM [file NIHMS1616338-supplement-Rigosi_JPD_SM.docx]

**Supplementary material: Analysing quantized resistance behaviour in graphene Corbino *p-n* junction devices**

Chieh-I Liu^1,2^, Dominick S. Scaletta^3^, Dinesh K. Patel^1,4^, Mattias Kruskopf^1,5,6^, Antonio Levy^1^, Heather M. Hill^1^, and Albert F. Rigosi^1^

^1^Physical Measurement Laboratory, National Institute of Standards and Technology (NIST), Gaithersburg, MD 20899, United States

^2^Department of Chemistry and Biochemistry, University of Maryland, College Park, MD 20742, United States

^3^Department of Physics, Mount San Jacinto College, Menifee, CA 92584, United States

^4^Department of Physics, National Taiwan University, Taipei 10617, Taiwan

^5^Joint Quantum Institute, University of Maryland, College Park, MD 20742, United States

^6^Electricity Division, Physikalisch-Technische Bundesanstalt, Braunschweig 38116, Germany

Table of Contents

1. A more direct *p-*type measurement

2. Analysis of the q_2_ formula

3. Device fabrication details

4. List of experimental data - *q*_1_ and *q*_2_

5. Monitoring the changes in carrier density

6. High-quality graphene growth

7. Shot noise and Fano factor calculation

8. Comparison of other Corbino devices

Keywords: quantum Hall effect, Corbino geometry, graphene *p-n* junctions

1. A more direct *p-*type measurement

As described in the main text, PMMA/MMA was deposited as a mediation layer for ZEP520A, a polymer with photoactive properties. This photoactivity enables graphene to become *p*-type upon exposure to an external ultraviolet lamp (254 nm). After exposure, typically of the order 10 hours (see section 3), the device is measured to determine the doping level. Fig. S1 shows an example control device fabricated under the same conditions, in parallel, as the Corbino devices. This traditional Hall bar with a *pn*J was measured across the central region, the intended *p-*type region, to show the electron (or hole, in this case) density after the exposure to the ultraviolet lamp. The data are shown in the inset, with the central region having a *p-*type doping of 2.5 × 10^11^ cm^-2^. The first region, intended for *n*-type doping, still exhibited doping close to the Dirac point, as seen by its Hall measurement appearing collapsed, indicative of electron and hole puddle formation.


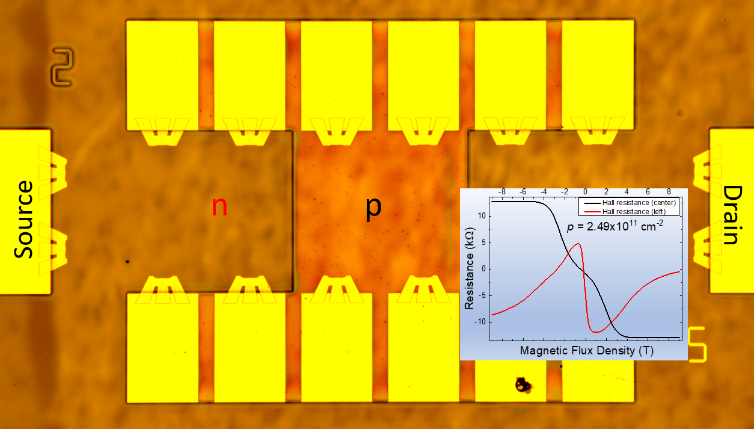


Fig. S1. Optical image of an example control device fabricated to determine the *p-*type doping obtained after exposure to an ultraviolet lamp. The exposure is repeatable, giving a gauge for the expected shift in any device fabricated with the same conditions.

2. Determining one of the terms of the *q_2_* formula

As described in the main text, for all cases in *N* = 3, the parameter $x_{0}=\frac{n_{1}+n_{2}}{n_{1}+n_{2}+1}$, and the general CER formula becomes:

$$q_{2}\left( n_{1},n_{2} \right)=\frac{q_{2}^{L}\left( M-n_{1}-n_{2} \right)+q_{2}^{(0)}x_{0}}{\left( M-n_{1}-n_{2} \right)+x_{0}}$$

(S1)

The difference between harmonized and discordant cases is embedded in the term $q_{2}^{(0)}$, which takes on the values $\frac{\left( n_{1}+1 \right)\left( n_{2}+1 \right)}{n_{1}+n_{2}}$ or $\frac{n_{1}+n_{2}+n_{1}n_{2}}{n_{1}+n_{2}}$, respectively. The values for $q_{2}^{(0)}$ were found empirically. For a given pair of $n_{1}$ and $n_{2}$, the analyses that were shown in the main text (that is, allowing $n_{x}\to\infty$ - see Fig. 3 (c) and Fig. 5 (a)) were repeated to obtain a single value of $q_{2}^{(0)}$. In most cases, the value was a fraction with a denominator equal to $n_{1}+n_{2}$. The numerator values appeared to alternate in a manner similar to that in Fig. 3 (a). The numerator values were plotted in Fig. S2 as a function of $n_{1}$ and $n_{2}$, with the green and white boxes representing harmonized and discordant cases.


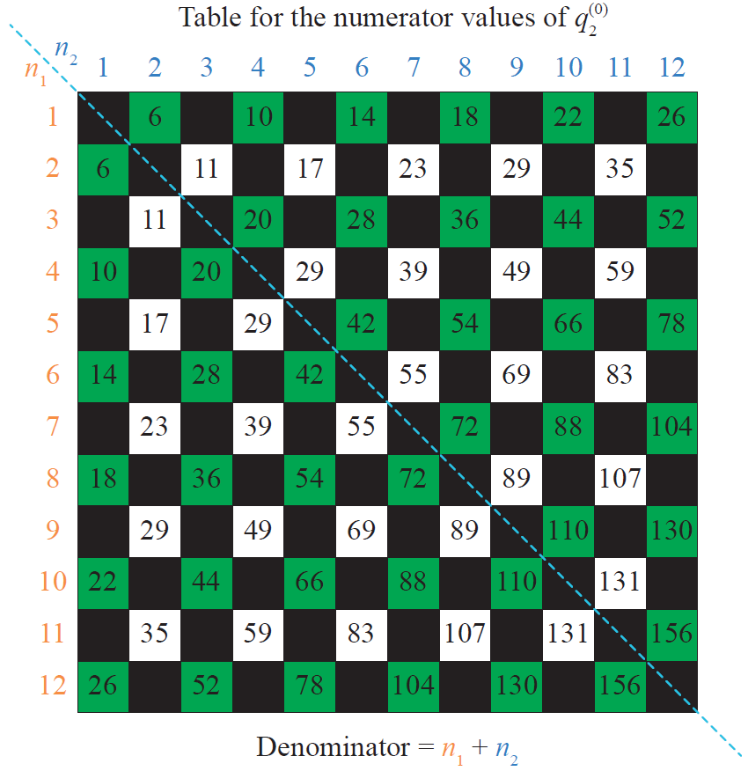


Fig. S2. Table of values for the numerator of $q_{2}^{(0)}$. Many simulations were performed to analyze the trends of this term, and in all cases, the denominator remained unchanged as $n_{1}+n_{2}$. The values in green and white reflect those found in the harmonized and discordant cases, respectively. The dotted blue line indicates an existing symmetry between $n_{1}$ and $n_{2}$. From this table, the two values listed above were found to generalize this trend.

3. Device fabrication details

Devices underwent additional fabrication steps after unmodified Corbino devices were functionalized with Cr(CO)_3_. The steps are listed below [see Notes]:

1. Spin photoresist at 523.6 rad/s for 1 min (acceleration: 523.6 rad/s^2^). Photoresist composed of 75 % electronic-grade propylene glycol monomethyl ether acetate, 15 % mixed cresol novolak resin, and 10 % diazo photoactive compound (sold commercially as S1813 from The Dow Chemical Company).

2. Bake chips at 115 °C for 1 min.

3. Expose to ultraviolet light (365 nm) for 5 seconds, using a photomask designed to expose only the central region of the device, as in Figure 4 (other designs shown later). Parameters were measured on a high-precision mask and bond aligner (sold commercially as the Suss MA6 from Suss Microtec). {1130 W; WEC = cont.; Vacuum mode: 10 s / 30 s / 15 s; Al-gap = 15 µm; Dosage = 100 mJ}.

4. Use solution of 97 % water, 2.3 % tetramethylammonium hydroxide, and 0.7 % polyglycol (commercially sold as MF-26A Developer from The Dow Chemical Company), for 1 min to remove exposed region.

5. Bake chips at 200 °C for 5 min.

6. Spin solution of 95 % ethyl lactate and 5 % Poly(methyl methacrylate/methacrylic acid) as a spacer layer (sold commercially as EL6 from The Dow Chemical Company). Thickness of this photoresist should be between 50 nm to 100 nm (or less). Spin rate is 523.6 rad/s (acceleration of 523.6 rad/s^2^).

7. Bake chips at 170 °C for 1 min.

8. Spin photoresist at 418.88 rad/s for 1 min to get a desired thickness between 300 nm and 400 nm. Photoresist composed of 89 % anisole and 11 % methyl styrene / chloromethyl acrylate copolymer (sold commercially as ZEP 520A from Zeon Chemicals L. P.).

9. Bake chips at 170 °C for 1 min.

10. Expose chips to 254 nm ultraviolet light for approximately 15 hours to activate the *p*-type regions. This time can be altered based on distance from the sample.

11. Other device regions should be inherently *n*-type. Level of doping can be adjusted by annealing.

4. List of experimental data - *q_1_* and *q_2_*

The experimental data graphically represented in Fig. 4 (b) and (c) of the main text is shown below:

*n*_1_ *q*_1_ (b) *q*_1_ (c)

0 16/16 17/16

1 31/16 32/16

2 44/16 45/16

3 55/16 56/16

4 64/16 65/16

5 71/16 72/16

6 76/16 77/16

7 79/16 80/16

8 80/16 81/16

9 79/16 80/16

10 76/16 77/16

11 71/16 72/16

12 64/16 65/16

13 55/16 56/16

14 44/16 45/16

15 31/16 32/16

The experimental data graphically represented as harmonized and discordant cases in Fig. 5 (b) and (c), respectively, of the main text is shown below:

*Case n*_1_ *n*_2_ *q*_12_

1 (b) 1 3 13/8

2 (b) 1 9 140/76

3 (b) 3 9 25/8

4 (b) 2 9 180/71

5 (b) 2 5 180/79

6 (b) 2 13 84/31

-- -- -- --

1 (c) 1 3 103/64

2 (c) 1 1 4/3

3 (c) 2 6 7/3

4 (c) 1 2 3/2

5 (c) 7 8 143/31

6 (c) 6 7 223/55

5. Monitoring the changes in carrier density

Evidence of gate-tuning effects on the devices are summarized here. An approximate method for montioring the carrier density was performed by measuring the bulk resistivity of an example pair of adjacent regions intended for both doping polarities. From reference [1], we know that *n*-type regions will be adequately protected from UV exposures, thus providing a stable bulk resistivity for the entire exposure time.

Fig. S3 (a) shows an optical image of an example Corbino *pn*J device. A two-terminal measurement for the bulk resistivity at room temperature was performed to approximately assign the time for when the *p*-type regions have been fully converted such that they would exhibit a flat ν = 2 plateau. The shaded blue region indicates the range of combined longitudinal resistivities of both regions that would not yield quantized plateaus. In Fig. S3 (b), the average bulk resistivity (of both regions) as a function of UV exposure time is shown here, with the changes attributable to the changes in the *p*-type region carrier densities. The behavior at the start of the exposure is from heating effects and the dip in the middle of the exposure (shaded red) results from the competing effects of heating and UV exposure [1]. The charge neutrality point (CNP) is labelled in gold to indicate when the carrier density begins to increase (*p*-type). The decrease of the bulk resistivity is an observation suggesting that the *p*-type region has been adjusted to at least 10^11^ cm^-2^, the approximately minimum value condition for observing a quantized ν = 2 plateau at low field strengths (2 T to 5 T) and cold temperatures (1.6 K).


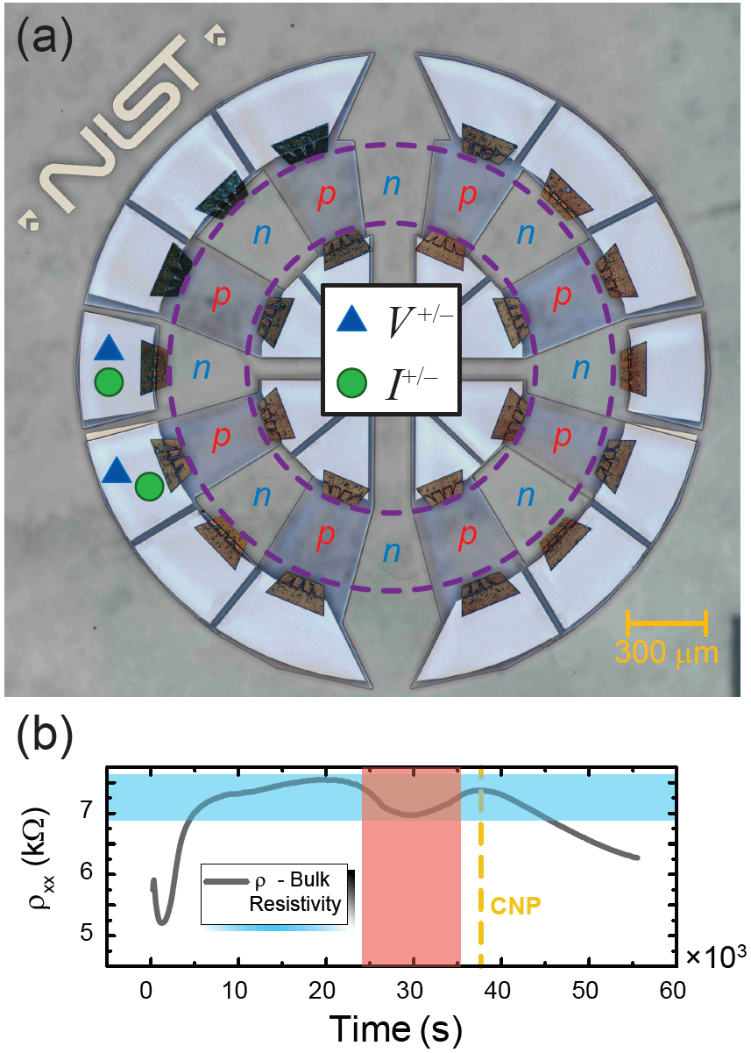


Fig. S3. (a) Optical image of an example Corbino *pn*J device. A two-terminal measurement for the bulk resistivity at room temperature is performed to gauge the point when the *p*-type regions have been fully converted. (b) The bulk resistivity (averaged over both the stable *n*-type region and the varying *p*-type region) as a function of UV exposure time is shown here, with the changes attributable to the changes in the *p*-type region carrier densities. The behavior at the start of the exposure is from heating effects and the dip in the middle of the exposure (shaded red) results from the competing effects of heating and UV exposure [1]. The charge neutrality point (CNP) is labelled in gold to indicate when the carrier density begins to increase (*p*-type).

One additional metric to be aware of for these epitaxial graphene devices is the mobility. From reference [2], adequate mobilities for epitaxial graphene devices can range from 1000 cm^2^V^-1^s^-1^ to 3000 cm^2^V^-1^s^-1^. However, for our devices, even by considering possible degradation of quality from the polymer interfaces, we measured mobilities between 3000 cm^2^V^-1^s^-1^ and 5000 cm^2^V^-1^s^-1^ for both *n*-type and *p-*type regions. These mobilities are obtained by calculating the slope (carrier density) from the counterpart devices in the first section and by using the observed bulk resistivities in Fig. S3 (b).

6. High-quality graphene growth

Evidence of the high-quality graphene growth is provided in this section. Though this growth method is covered heavily in other work [3], it would be beneficial to provide measurements that support the method’s reproducibility. Before functionalizing any Corbino graphene devices, both Raman spectral maps and atomic force microscopy (AFM) images were acquired to verify that the homogenous material was single-layer graphene (see Fig. S4). The Raman map in Fig. S4 (b) shows a scatterplot of the position and width of the 2D (G’) mode in graphene.


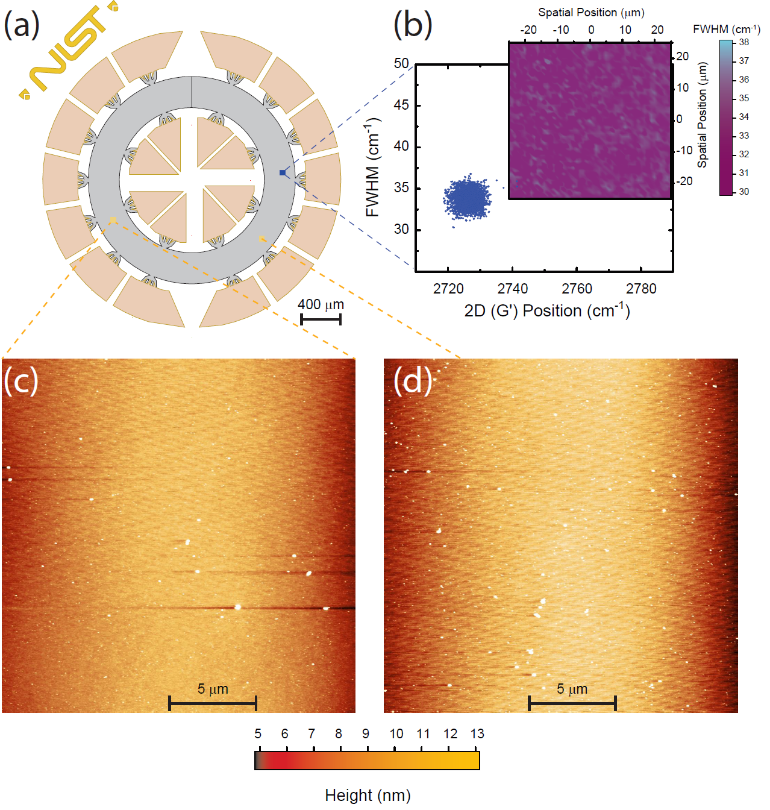


Fig. S4. (a) Schematic of an example Corbino *pn*J device. (b) Raman map taken at the approximate location of the blue square in (a), with a 50 μm range in both lateral directions. (c) and (d) AFM images of two different locations on the device were acquired to support the homogeneity of the growth.

Square Raman maps were collected with step sizes of 1 μm in a 50 by 50 raster-style grid and were acquired with a Renishaw InVia micro-Raman spectrometer^[see notes]^ using a 633 nm wavelength excitation laser source and a backscattering configuration. The spot size was approximately 1 µm, the acquisition times were 300 s, the laser power was 1.7 mW power, and the optical path included a 50 × objective and 1200 mm^-1^ grating. The spread in Fig. S4 (b) is indicative of a relatively homogeneous sample. AFM images (Fig. S4 (c) and (d)) were acquired with using an Asylum Cypher^[see notes]^ in tapping mode (1 Hz) at two regions of the device.

7. Shot noise and Fano factor calculation

Additional details regarding the shot noise and Fano factor are provided here, using Ref. [4] as a supplementary guide. The Fano factor is calculated by the following:

$$F=\frac{S_{I}}{2eI}=\frac{1}{2eI}\left( 2eG_{0}V_{sd} \right)\frac{\left( \left| \nu_{1} \right|\left| \nu_{2} \right| \right)^{2}}{\left( \left| \nu_{1} \right|+\left| \nu_{2} \right| \right)^{3}}=0.25$$

(S2)

In Eq. S2, S_I_ is the shot noise at zero temperature and assumes a double-step energy distribution [4]. However, when we consider the case of the used current (~10 mV), Fano factor from experimental results in [4] (*F* = 0.015), and temperature broadening [5]:

$$\Delta S_{I}=2eF\left( G_{pnJ}V_{sd} \right)\left[ \coth\left( \frac{eV_{sd}}{2k_{B}T} \right)-\frac{2k_{B}T}{eV_{sd}} \right]$$

(S3)

In Eq. S3, $\Delta S_{I}$ is the shot noise difference (or excess shot noise) obtained by substracting the noise at the Dirac point and adjusting the source-drain voltage. The main finding in [4] was that the shot noise amplitude (and thus the Fano factor) was reduced to a negligible value on mesoscopic (~10 μm) length scales. Furthermore, the cause of the amplitude decrease is attributable to energy relaxation processes along the junction [4].

8. Comparison of other Corbino devices

Additional details regarding other Corbino graphene *pn*J devices are included here to give advantages and disadvantages in the context of the applications listed in the introduction of the main text.

| Material | Processing time from raw material to *pn*J device | Lateral scales | Max Current |
| --- | --- | --- | --- |
| Epitaxial – Ref. [6] | 7 hr – 12 hr | > 1 mm | 770 μA |
| Exfoliated – Ref. [7] | 6 hr – 9 hr | 100 μm | 10 μA |
| Exfoliated – Ref. [8] | 15 hr – 20 hr | 100 μm | 25 μA |

Overall, the major advantages of the graphene Corbino *pn*Js are that scaling to millimetre order is relatively simple, as are the corresponding fabrication steps. For instance, using electron beam lithography can take a significantly longer time (of the order a few hours of tool time) than standard ultraviolet lithography (of the order a few minutes of tool time). The disadvantages include elements like not being able to bottom-gate due to the insulating nature of SiC and only being able to access the quantized ν = 2 plateau for either polarity, which is also due to the SiC/buffer layer interface. Overall, there are applications that may benefit from the processes and framework contained in the main text.

Notes

Commercial equipment, instruments, and materials are identified in this paper in order to specify the experimental procedure adequately. Such identification is not intended to imply recommendation or endorsement by the National Institute of Standards and Technology or the United States government, nor is it intended to imply that the materials or equipment identified are necessarily the best available for the purpose.

References

1. Rigosi AF, Patel DK, Marzano M, Kruskopf M, Hill HM, Jin H, Hu J, Hight Walker AR, Ortolano M, Callegaro L, Liang CT and Newell DB 2019 *Carbon* **154** 230
2. Fukuyama Y, Elmquist RE, Huang LI, Yang Y, Liu F-H and Kaneko NH 2015 *IEEE Trans. Instrum. Meas*. **64** 1451
3. Kruskopf M, Pakdehi DM, Pierz K, Wundrack S, Stosch R, Dziomba T, Götz M, Baringhaus J, Aprojanz J and Tegenkamp C 2016 *2D Mater*. **3** 041002
4. Kumada N, Parmentier FD, Hibino H, Glattli DC, Roulleau P 2015 *Nat. Commun.* **6** 8068
5. Blanter Y M and Buttiker M 2000 *Phys. Rep*. **336** 1
6. Kruskopf M, Rigosi AF, Panna AR, Patel DK, Jin H, Marzano M, Berilla M, Newell DB, Elmquist RE 2019 *IEEE Trans. Electron Dev.* **66** 3973
7. Yanık C, Kaya II 2013 *Solid State Commun.* **160** 47
8. Hu J, Rigosi AF, Kruskopf M, Yang Y, Wu BY, Tian J, Panna AR, Lee HY, Payagala SU, Jones GR, Kraft ME, Jarrett DG, Watanabe K, Taniguchi T, Elmquist RE and Newell DB 2018 *Sci. Rep*. **8** 15018
